# An Aerial–Ground Robotic System for Navigation and Obstacle Mapping in Large Outdoor Areas

**DOI:** 10.3390/s130101247

**Published:** 2013-01-21

**Authors:** Mario Garzón, João Valente, David Zapata, Antonio Barrientos

**Affiliations:** Centro De Automática y Robótica, UPM-CSIC. Calle José Gutiérrez Abascal, 2. 28006 Madrid, Spain; E-Mails: joao.valente@upm.es (J.V.); david.zapata@etsii.upm.es (D.Z.); antonio.barrientos@upm.es (A.B.)

**Keywords:** autonomous navigation, cooperative perception, obstacle avoidance, outdoor mapping, collaborative robotics, UGV, UAV

## Abstract

There are many outdoor robotic applications where a robot must reach a goal position or explore an area without previous knowledge of the environment around it. Additionally, other applications (like path planning) require the use of known maps or previous information of the environment. This work presents a system composed by a terrestrial and an aerial robot that cooperate and share sensor information in order to address those requirements. The ground robot is able to navigate in an unknown large environment aided by visual feedback from a camera on board the aerial robot. At the same time, the obstacles are mapped in real-time by putting together the information from the camera and the positioning system of the ground robot. A set of experiments were carried out with the purpose of verifying the system applicability. The experiments were performed in a simulation environment and outdoor with a medium-sized ground robot and a mini quad-rotor. The proposed robotic system shows outstanding results in simultaneous navigation and mapping applications in large outdoor environments.

## 1. Introduction

The navigation of a mobile robot can be described as the problem of finding a suitable and collision-free motion between the two poses (position and orientation) of this robot, while obstacle mapping consists of using the sensing capabilities to obtain the representation of unknown obstacles in such a manner that it is useful for navigation [1]. This work presents an alternative to solve these two problems by using a system that combines an aerial and a ground robot, using only the localization system of the terrestrial robot (UGV) and the image from a single camera on board the aerial robot (UAV).

The localization and obstacle detection techniques that are needed for ground robot navigation systems can use relative or absolute sensors. Relative sensors refers to those that are usually internal to the robot, and its measurement is given with respect to another position or state. Absolute sensors provides a measurement with respect to an external or global reference frame [2]. The idea presented here is to provide a vision system on board the aerial robot as a relative sensor, and fuse this information with the absolute position of the ground robot in order to achieve an absolute measurement of the position of the obstacles. By doing this it is possible to obtain a mechanism of collaborative navigation and obstacle avoidance, that takes advantage of the heterogeneity of a mixed robotic system.

There are two main objectives for this system: The first is to obtain the relative distance from the UGV to any unknown obstacle surrounding it, and in that way, guarantee the safe navigation towards the goal or way-point. The second one is to obtain the absolute position of all obstacles detected during the navigation and build a map with their localization; this means having a list of the geo-referenced position of the obstacles. Some previous works have used a combination of aerial and ground robots to approach similar objectives [3–5], but there is no previous work that would have used only one on-board aerial camera for obstacle detection and avoidance; also, to our knowledge, no previous obstacle mapping was performed with fusion of an aerial image and the localization of the UGV.

One of the advantages of using an aerial visual navigation system is that the UGV field of view (FOV) is dynamic. This means that by controlling the UAV height and relative position, the UGV at some stage can perform a pseudo-zoom on an obstacle or any interesting object, as well as reconnaissance of areas outside the UGV's FOV. Moreover, it is also possible to identify rugged terrain, floor openings or negative obstacles, and other unexpected navigation obstructions in the UGV surrounds. This collaboration ensures the UGV's safety while it performs other inspection tasks. Finally, the system does not require previous knowledge from the environment and it can cover large navigation perimeters.

The collision-free navigation system is being developed in several phases. In the first step, the UGV and the obstacles in the robot pathway are identified by processing the aerial image, then techniques based in potential fields are applied to enable simple navigation and obstacle avoidance. After that, the proposed architecture will allow a local map to be built and obtain a geo-referenced position of the obstacle seen by the UAV. Also, each obstacle found can be memorized, and a global map with all the obstacles can be built. This will enable the system to merge reactive and deliberative methods.

The outline of this article is as follows: After this brief introduction, Section 2 reviews some related works. Section 3 introduces the addressed challenges. The techniques used to estimate the UGV's pose and the obstacle position are described in Section 4. Then, in Section 5, the UGV navigation and control systems used are explained. After that, the experiments performed and the results obtained both in simulations and with real robots are presented in Section 6. Finally, the conclusions are presented.

## 2. Related Work

Mobile robotic systems, both aerial and terrestrial, have been studied and developed over the years for several civil and military purposes. Some of those applications are focused on using mobile robots to help or substitute humans in tedious or dangerous tasks, as well as to survey and patrol large unstructured environments. This task is one of the objectives of the ROTOS project in which this work is framed.

In order to perform perimeter surveillance, a robot must be able to generate a trajectory to explore, or to navigate from, an initial point to a final point without colliding with other vehicles or obstacles. This autonomous navigation is one of the most ambitious issues in robotics research.

Concerning visual navigation, many reactive and deliberative navigation approaches have been presented up to now, e.g., in structured environments using white line recognition [6], in corridor navigation using View-Sequenced Route Representation [7], or more complex techniques combining visual localization with the extraction of valid planar region [8], or visual and navigation techniques to perform visual navigation and obstacle avoidance [9]. Some works integrate and fuse vision data from the UAV and UGV for best target tracking [3], another work [10] presents uncertainty modeling and observation–fusion approaches that produce considerable improvement in geo-location accuracy. Also, a different work [11] presents a comparative study of several target estimation and motion-planning techniques and remarks on the importance (and the difficulty) of a single UAV at maintaining consistent view of moving targets.

By merging all those capacities and characteristics together, it is possible to develop a unique sensing and perception of a collaborative system. Work [4] focus their work on a multi-robot system based on a vision-guide autonomy quad-rotor. They describe a way to take off, land and track over the UGV, where the UGV is equipped with two LEDs and a flat pattern in the surface. However, the quad-rotor does not provide information to the UGV about the environment. On the other hand, work [12] presents a motion-planning and control system based on visual servoing (*i.e.*, the use of feedback from a camera or a network of cameras) in a UGV without cameras on board, but not specifically with a UAV. Even work [5] proposes a hierarchy in which several UAVs with aerial cameras can be used not only to monitor, but also to command a swarm of UGVs.

Some of those studies have been tested in several different contexts, such as environment monitoring [13], pursuit-evasion games [14], fire detection and fighting [15], multi-robot localization in highly cluttered urban environments [16], de-mining [17], and other multi-purpose collaborative tasks [18].

## 3. Problem Formulation and Solving

In this section, we introduce the problem from a theoretical perspective. In the first place, a geometric description of the problem, as well as the relationships between the different reference frames, are given. Then, a solution is proposed based on the aerial–ground system kinematics and some assumptions. As mentioned before, there are two main objectives of the aerial–ground system:

1. **Obstacle avoidance:** provide collision-free navigation for the UGV during the execution of its mission through visual feedback from a low cost mini UAV.
2. **Obstacle mapping:** obtaining the global position of the obstacles and built a geo-referenced map from it.

### 3.1. Coordinate Frames and Definitions

**Figure 1.**
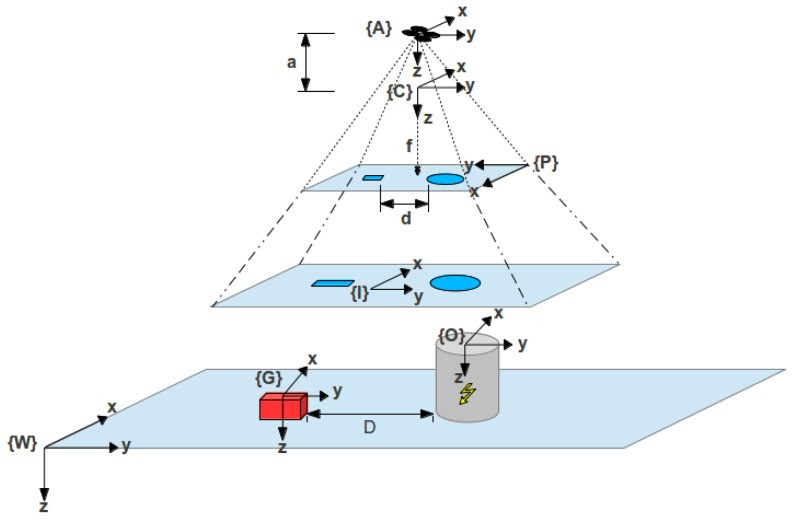
Coordinate frames.

In the first place, the coordinate systems are defined using the right-hand rule. This step is important and should not be avoided since those definitions are used in the development of the guidance, navigation and control (GNC) sub-system. The coordinate frames from all physical bodies within the workspace are defined with the right-hand rule. The reference frames adopted by the aerial–ground robotic system are shown in Figure 1.

The nomenclature used in the coordinate systems is the follow: W (world frame), G (ground frame), O (Obstacle frame), I (image plane), P (pixels plane), C (camera frame), and A (aerial frame). All the coordinate frames are defined as three dimensional (3*D*), with the exception of the image plane and the pixels plane, which are two-dimensional (2*D*).

In this work, some assumptions have been considered. The goal is that the UAV follows the UGV and hovers at a minimum height above it. This minimum height must ensure that UGV is always within the camera FOV (field of view (FOV)), likewise the UGV surroundings. In this way, any obstacle nearby can easily be identified. Therefore, it is assumed that the UAV has a GNC (guidance, navigation and control) sub-system that enables it to follow and hover above UGV. Moreover, one single obstacle is identified at a time in the camera FOV.

### 3.2. Sensor Geometric Model

The sensor model can be mapped by a physical model (more complex, by considering highly non-linear equations) or analytical (approximated, polynomial functions). Additionally, the sensor model, *i.e.*, the digital camera, can be expressed either forward (image to ground) or inversely (ground to image). In this work, the forward physical model is considered. Let us start from the pinhole camera model from Equation (1), where (*u*, *υ*, 1)*^T^* is a 2D point in the pixel frame and (*X*, *Y*, *Z*, 1)*^T^* a 3D point in the workspace of the robot. The perspective projection is scaled by a factor *s*.

(1) $$\underset{s\times p}{\underset{︸}{s\times \left[\begin{array}{l} u\\ \upsilon \\ 1 \end{array}\right]}}=\underset{K}{\underset{︸}{\left[\begin{array}{ccc} f_{x} & 0 & c_{x}\\ 0 & f_{y} & c_{y}\\ 0 & 0 & 1 \end{array}\right]}}\;\underset{\left[R|T\right]}{\underset{︸}{\left[\begin{array}{cccc} r_{11} & r_{12} & r_{13} & t1\\ r_{21} & r_{22} & r_{23} & t2\\ r_{31} & r_{32} & r_{33} & t3 \end{array}\right]}}\;\underset{P}{\underset{︸}{\left[\begin{array}{c} X\\ Y\\ Z\\ 1 \end{array}\right]}}$$

#### 3.2.1. Intrinsic Parameters

The intrinsic parameters of the camera are addressed by the first matrix from Equation (1), denoted as *K*. With these parameters, a relationship can be established between the pixel coordinates in the image and its position in the camera reference frame. The intrinsic parameters of interest (known *a priori* from the camera specifications) are: *f*, focal length of the camera; *c_x_* and *c_y_*, which are the principal points of the image (*i.e.*, pixel coordinates definition from the center) and lens distortion coefficients (explained later ahead in Section 3.2.3.).

#### 3.2.2. Extrinsic Parameters

The extrinsic parameters, on the other hand, define the camera position and orientation with reference to the world reference system. The obstacles position within the world coordinate frame can be obtained in two ways:

1. Obtaining the obstacle position in the world coordinate frame and apply all geometric transformations (*i.e.*, forward camera model) from the pixel plane to the world reference frame.
2. Obtain the distance from the UGV to the obstacle in meters in the image plane, and compute the obstacle position in the world reference frame from the UGV position and orientation in the world reference frame (given by a GPS). Herein, there is no need to translate the coordinates to the world frame.

The proposed approach employs the methodologies described in the item 2. Thus, the only extrinsic parameter employed and necessary is the orientation from the camera which ensures geometric corrections on the image acquired by the camera shipped on the UAV.

From Figure 1, it can be noticed that the camera reference frame is displaced from the camera reference frame over the z-axis. This displacement represents the distance from the camera to the UAV center of gravity (CoG). The camera position with relation to the UAV can be expressed as

(2) $$\left[\begin{array}{c} {\text{x}}_{C}^{A}\\ {\text{y}}_{C}^{A}\\ {\text{z}}_{C}^{A} \end{array}\right]=\left[\begin{array}{c} x_{A}\\ y_{A}\\ z_{A} \end{array}\right]+\left[\begin{array}{c} 0\\ 0\\ a \end{array}\right]$$

Furthermore, the UAV position is already given in world coordinates (*i.e.*, provided by the GPS). Thus, the camera position in the world reference frame is given again by Equation (2).

The position and heading from the UGV and the obstacle position in the image reference frame are extracted and projected in the pixel frame by applying computer vision algorithms and techniques. The obstacle position or the UGV position can be obtained by applying this simple perspective projection in Equation (3), its relative position to each other, obtained by a simple subtraction, can be used when the navigation is purely reactive.

(3) $$\left[\begin{array}{c} {\text{x}}_{O}^{I}\\ {\text{y}}_{O}^{I} \end{array}\right]=\left[\begin{array}{ccc} f_{x} & 0 & c_{x}\\ 0 & f_{y} & c_{y} \end{array}\right]\;\left[\begin{array}{ccc} \cos\;(\theta ) & \sin\;(\phi )\sin\;(\theta ) & \cos\;(\phi )\sin\;(\theta )\\ 0 & \cos\;(\phi ) & -\mathrm{sen}\;(\phi )\\ -\sin\;(\theta ) & \sin\;(\phi )\cos\;(\theta ) & \cos\;(\phi )\cos\;(\theta ) \end{array}\right]\;\left[\begin{array}{c} x_{p}z_{A}\\ y_{p}z_{A}\\ 1 \end{array}\right]$$

**Figure 2.**
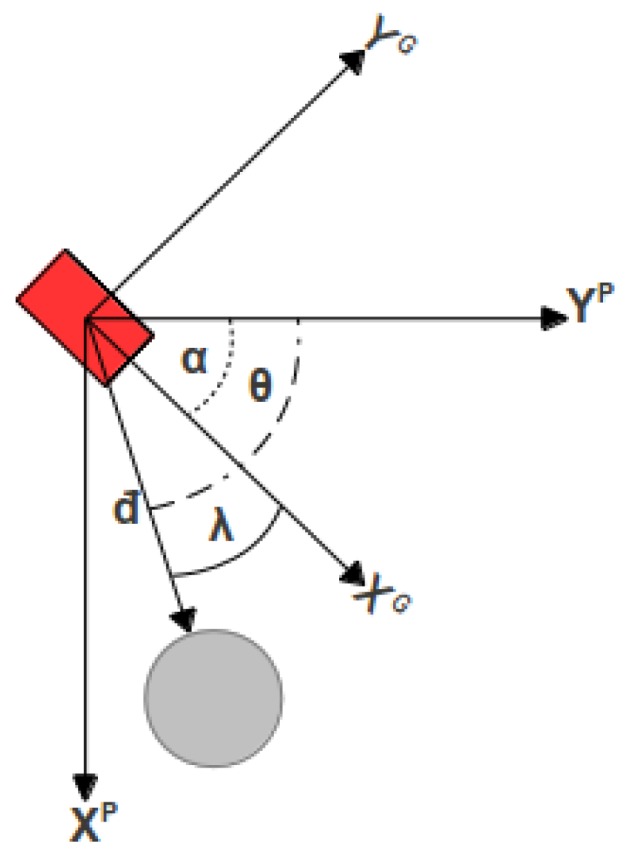
UGV heading geometry. The angle of interest is given by *γ* = *θ* − *α*.

The angle between the obstacle and the UGV in the UGV's reference frame, denoted as *γ*, can be obtained by subtracting the UGV heading in the camera plane denoted as *α* from the angle of the obstacle, also on the camera plane and denoted as *θ*. Both of those angles can be obtained by using Equation (3). This is better illustrated in Figure 2.

Finally, the last objective of the system is to build a global map with all obstacles found in the path of the UGV. Each obstacle identified by the UAV camera is geo-referenced and targeted in a geo-referenced map. Thus, the obstacle position in the world coordinate frame is given by

(4) $$\left[\begin{array}{c} {\text{x}}_{O}^{W}\\ {\text{y}}_{O}^{W}\\ {\text{z}}_{O}^{W} \end{array}\right]\;=\;\left[\begin{array}{ccc} \cos\;(\gamma ) & -\sin\;(\gamma ) & 0\\ \sin\;(\gamma ) & \cos\;(\gamma ) & 0\\ 0 & 0 & 1 \end{array}\right]\;\left[\begin{array}{c} {\text{x}}_{O}^{I}-{\text{x}}_{G}^{I}\\ {\text{y}}_{O}^{I}-{\text{y}}_{G}^{I}\\ {\text{z}}_{O}^{I}-{\text{z}}_{G}^{I} \end{array}\right]\;+\;\left[\begin{array}{c} {\text{x}}_{G}^{W}\\ {\text{y}}_{G}^{W}\\ {\text{z}}_{G}^{W} \end{array}\right]$$

#### 3.2.3. Lens Distortions

Additionally, the Brown-Conrady model is applied to rectify the lens distortion. The lens distortion model is given by two distortion coefficients: the radial (*k*_1_,…, *k_n_*) and the tangential (*d*_1_,…, *d_n_*). Thus, considering ${\left({\text{x}}_{p}^{\prime },{\text{y}}_{p}^{\prime }\right)}^{T}$, an undistorted image point, and (*x_p_*, *y_p_*)*^T^*, a distorted image point, the rectified equations are given by,

(5) $$\left[\begin{array}{c} x_{p}\\ y_{p} \end{array}\right]=\left[\begin{array}{c} {\text{x}}_{p}^{\prime }(1+k_{1}r^{2}+k_{2}r^{4})+2d_{1}+2p_{1}{\text{x}}_{p}^{\prime }{\text{y}}_{p}^{\prime }+p_{2}(r^{2}+2x_{p}^{\prime 2})\\ y_{p}^{\prime }(1+k_{1}r^{2}+k_{2}r^{4})+p_{1}(r^{2}+2x_{p}^{\prime 2})+2p_{2}x_{p}^{\prime }y_{p}^{\prime } \end{array}\right]$$

### 3.3. Features Extraction

**Figure 3.**
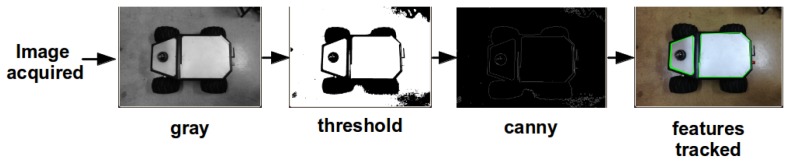
Features extraction procedure example for a ground robot.

As was already mentioned, the features extracted with the camera shipped on the UAV were obtained by applying computer vision techniques. The UGV position and orientation in the pixel plane is obtained through the pseudo-code shown in Algorithm 1. In this case, a polygon is defined as a geometric shape with more than three vertices. This procedure is accomplished by finding in the image processed a set of contours addressing the ground robot shape or any good polygonal feature addressing the robot. The image processing sequence is exemplified in Figure 3. A threshold is applied to a gray scale image, followed by a canny edge detector. Once the polygon is identified, its centroid can be easily obtained. Moreover, the UGV orientation relative to the image is obtained through the contour principal moments of inertia.


**Algorithm 1** UGV pose extraction.
 1:*Countors* ← *FindCountours*(*Image*_<_*_gray_*_>_) 2:**if** 3 < *length*(*Countors*) **then** 3: *P* ← *GetPolygn*(*Contours*) 4: *Point*_<_*_px,Py_*_>_ ← *Centroid*(*P*) 5:**end if** 6:*M* ← *getMomentums*(*P*) 7:*α* ← *Angle*(*M*) 8:
$\left({\text{x}}_{G}^{P},{\text{y}}_{G}^{P})\leftarrow \text{getCentroid}(P\right)$


Concerning obstacles, those with round and square shape (e.g., silos, water or gas tanks, containers) have been considered for identification in this work. The square features are extracted similarly to the aforementioned procedure. However, the round top shapes are featured, employing Hough transformations. It should be also highlighted that the obstacle is identified from its top view, since the camera is pointing downwards (see pseudo-code in Algorithm 2).


**Algorithm 2** Obstacle position extraction.
 1:*C* ← *HoughCircles*(*Image*_<_*_gray_*_>_) 2:
$\left({\text{x}}_{O}^{P},{\text{y}}_{O}^{P})\leftarrow \text{Centroid}(C\right)$ 3:*r* ← *Radius*(*C*)


Before the experiments, some parameters must be tuned manually, as, for example, the threshold, which must be regulated according to weather conditions, in particular, light exposure.

## 4. Position Estimation

This section describes the techniques used to estimate the UGV's pose and the obstacle position. First the UGV's pose in a global coordinate frame is estimated by fusing the odometry, IMU and GPS readings using an Extended Kalman Filter (EKF), after that, another Kalman Filter is used to estimate the position of the obstacles using the results of the image processing algorithm.

### 4.1. Ground Robot Pose Estimation

The vehicle pose estimation problem can be defined as the calculations necessary to estimate the vehicle state based on the readings from several sensors. This problem is solved by using an EKF, which produces at time *k* a minimum mean squared error estimate **ŝ**(*k* ∣ *k*) of a state vector **s**(*k*). This estimation is the result of fusing a prediction of the state estimate **ŝ**(*k* ∣ *k* − 1) with an observation **z**(*k*) of the state vector **s**(*k*).

- Vehicle Model
  The vehicle model represents its three-dimensional pose (position and orientation). This pose can be parametrized as **s** = [*t*, ψ]*^T^* = [*x*, *y*, *z*, *φ*, *θ*, *ψ*]*^T^*, where t = [*x*, *y*, *z*]^*T*^ are the Universal Transverse Mercator (UTM) coordinates and the relative height of the vehicle, and **Ψ** = [*φ*, *θ*, *ψ*]*^T^* are the Euler angles on the *X*-*Y*-*Z* axis also known as *Roll, Pitch, Yaw*.
  In order to use an EKF to estimate the pose of the robot, it is necessary to express it as Multivariate Gaussian Distribution **s** ∼ *N* (*μ*, Σ). This distribution is defined by a six-element column vector *μ*, representing the mean values. Moreover, a six-by-six symmetric matrix Σ represents the covariance.
  (6) $$\sum =\begin{array}{c} \mu ={\left[E[x],E[y],E[z],\;E[\varphi ],E[\theta ],E[\psi ]\right]}^{T}\\ \left[\begin{array}{cccc} \text{Cov}[x,x] & \text{Cov}[x,y] & \cdots & \text{Cov}[x,\psi ]\\ \text{Cov}[y,x] & \text{Cov}[y,y] & \cdots & \text{Cov}[y,\psi ]\\ \vdots & \vdots & \ddots & \vdots \\ \text{Cov}[\psi ,x] & \text{Cov}[\psi ,y] & \cdots & \text{Cov}[\psi ,\psi ] \end{array}\right] \end{array}$$
  Now it is necessary to define a non-linear conditional probability density function *f* (**s** (*k*), **u** (*k* + 1)) which represents the probability of the predicted position given the current vector state **s**(*k*) and the control vector **u**(*k* + 1).
  (7) s(k+1)=f(s(k),u(k+1))
  It is also very useful to define a global UGV transformation *T*_UGV_ consisting of a rotation matrix *R*(Ψ) obtained from the Euler angles, and a translation *t* in the global reference frame.
  (8) $$T_{\text{UGV}}=\left[R(\Psi )\;t\right]$$
- Measurement Models
  The vehicle pose is updated according to the readings from three different sensors: The internal odometry of the UGV, a IMU sensor and a GPS. Thus, it is necessary to create a model that links the measurements of each sensor with the global position of the mobile robot. As was done with the pose of the vehicle, the observations of the sensors need to be expressed as Gaussian Distribution; this means that they should provide a vector of mean values and a symmetrical matrix of covariances.
  As it was done for the estimated UGV pose, a transformation *T_i_* is defined for each measurement. These transformations are:
  – Odometry
    The odometry provides a relative position and orientation constraint. To be able to use these measurements in a global reference frame, the transformation between two successive readings of the odometry (*T_odom_*_−_*_read_*(*k*) → *T_odom_*_−_*_read_*(*k*+1)) is calculated, and the resulting transformation is applied to the previous estimated position of the UGV.
    (9) $$T_{\text{odom}}\;(k+1)=T_{\text{UGV}}\;(k)*T_{\text{odom}-\text{read}}\;{\left(k\right)}^{-1}*T_{\text{odom}-\text{read}}\;(k+1)$$
  – GPS.
    The readings from the GPS are converted to UTM using the equations form *USGS Bulletin 1532* [19], and they are used to provide a global position constraint. However, the GPS does not provide information for the orientation of the robot, so only the position should be taken into account.
    (10) $$T_{\text{GPS}}(k+1)=\left[R(\Psi_{o})\;t_{\text{GPS}-\text{READ}}(k+1)\right]$$
  – IMU.
    The IMU readings are pre-processed to fuse the gyroscopes, accelerometers and magnetometers. In order to provide a global constraint of the orientation of the UGV, no position or translation constraints are obtained from this sensor.
    (11) $$T_{\text{IMU}}(k+1)=\left[R(\Psi_{\text{IMU}-\text{READ}})\;t_{o}\right]$$
  With the transformations defined, it is possible to define a probability density function for each one of them. In all cases, the *H* matrix (necessary to compute the estimation of the EKF) will be an identity matrix of dimension six. For the GPS and the IMU, the covariance associated with the rotation or translation, which are not provided, will be replaced by very high values.

These models and the Kalman Filter itself are implemented using the Bayesian Filter Library [20], which is fully integrated in the ROS environment. The estimated pose is updated at a pre-defined frequency with the data available at that time; this is an estimation of the pose to be made even if one sensor stops sending information. Also, if the information is received after a time-out, it is disregarded.

**Figure 4.**
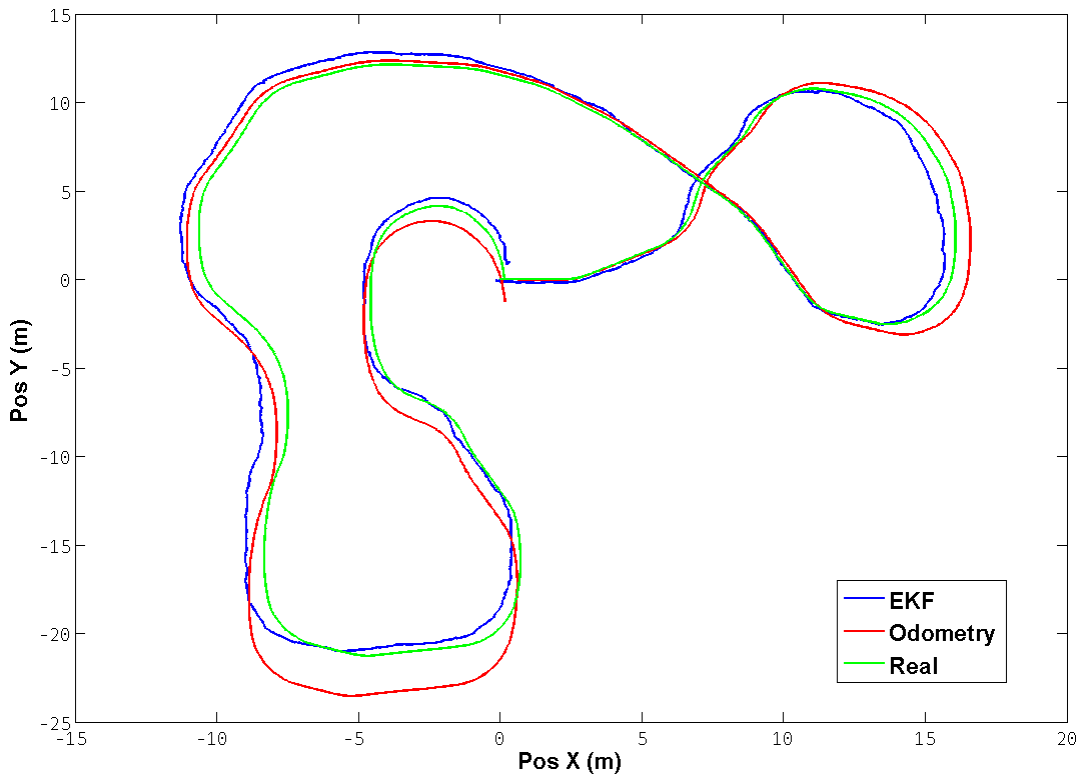
Test Trajectory for EKF. A trajectory was performed in order to test the performance of the Extended Kalman Filter.

Figure 4 shows a test trajectory performed with the robot in a simulated environment. The positions were translated to a common reference frame in order to be able to compare them. It can be observed that the EKF performs a correction of the position from the odometry, thereby reducing the mean square error in comparison with the real position obtained directly from the simulator.

### 4.2. Transformations and Obstacle Pose Estimation

Once the pose of the UGV is estimated, it is possible to apply the processing and transformations described in Section 3 to obtain the global position of the obstacle. For each image processed, a transformation like the one described on Equation (4) is obtained, and it is applied to the estimated position of the robot. This produces several measurements of the position of each obstacle detected. Since the global position of the UGV is obtained in UTM coordinates, the global position of the obstacles is given in the same reference frame. All of the positions are stored and then the mean value and the standard deviation are calculated. Finally. a table with the mean values and standard deviations is produced.

## 5. Navigation and Control

This section describes the algorithms and control laws used for the navigation of both the ground and aerial robots. Since this work is not oriented to find new control theories, a well-studied potential fields navigation is used to control the ground robot, and the aerial robot works in teleoperated mode.

### 5.1. Aerial Robot

The main objective of the aerial robot is to have the ground robot and as much as possible of its pathway in the field of view of its downward camera. This is assured by involving a human pilot who controls the air robot in teleoperated mode. However, there is a mid-level control system working to facilitate the operation of the air robot. The mid-level control system provides a height control based on the readings of a ultra sound range meter, it also provides a pose stabilization algorithm that attempts to keep the robot as steady as possible in order to obtain a better image, this stabilization technique is based on a fusion of optical flow and IMU readings. Since the dynamics of the aerial robot are much faster than the ground robot, there are several time lapses where the aerial robot is hovering; in those moments, the stabilization algorithm proves its utility.

Alternatively, it is possible to control the aerial robot using a ground control station (GCS) that receives the telemetry and images from the quad-rotor, before using a position control algorithm to maintain the target (UGV) in the center of the image, and sending commands to control the aerial robot flight motions (Roll *ψ* and Pitch *θ* angles). These control commands are sent to the aerial robot usually 30 times per second to obtain smooth movements. Additionally, the GCS is used for configuring and monitoring the aerial robot (see [21] for a detailed architecture).

### 5.2. Ground Robot

The ground robot is controlled by using a ROS implementation of the Steering Behaviors [22]. This potential fields navigation technique is based on the definition of a simple vehicle model, which is a point mass approximation. The vehicle is described by its position, orientation and velocity. Its velocity is modified by applying forces or accelerations that are limited by a maximum force parameter. This allows for a simple computational model to operate and is independent from the locomotion schema.

The pose of the vehicle is defined by four vectors, the first one is for the position of the robot, and the other three are ortho-normal vectors that define orientation (*Forward*, *Side* and *Up*), having the *X* axis as the normalized direction of the velocity or *Forward* vector. Since only planar movement is considered the *Z* axis will be constant, and the *Y* will be the cross product of *X* and *Z* (or *Forward* and *Up*). The control signal obtained from the algorithm is one vector that represents the steering force resulting from all the behaviors (*i.e.*, evasion, seeking, alignment). This vector is added to the current velocity and then is mapped in order to be translated to the vehicle reference frame. It is subsequently converted into two control signals—linear and angular velocity—that are sent to the ground robot control system.

The use of the steering behaviors library for the navigation of the UGV has two main advantages: it allows the implementation of several behaviors for a single UGV or a set of them, and it is possible to dynamically add or remove obstacles, which is very convenient in this case. For this initial effort, three steering behaviors were implemented: one for seeking the target point, another for arriving at it, and the third one to avoid obstacles.

**Figure 5.**
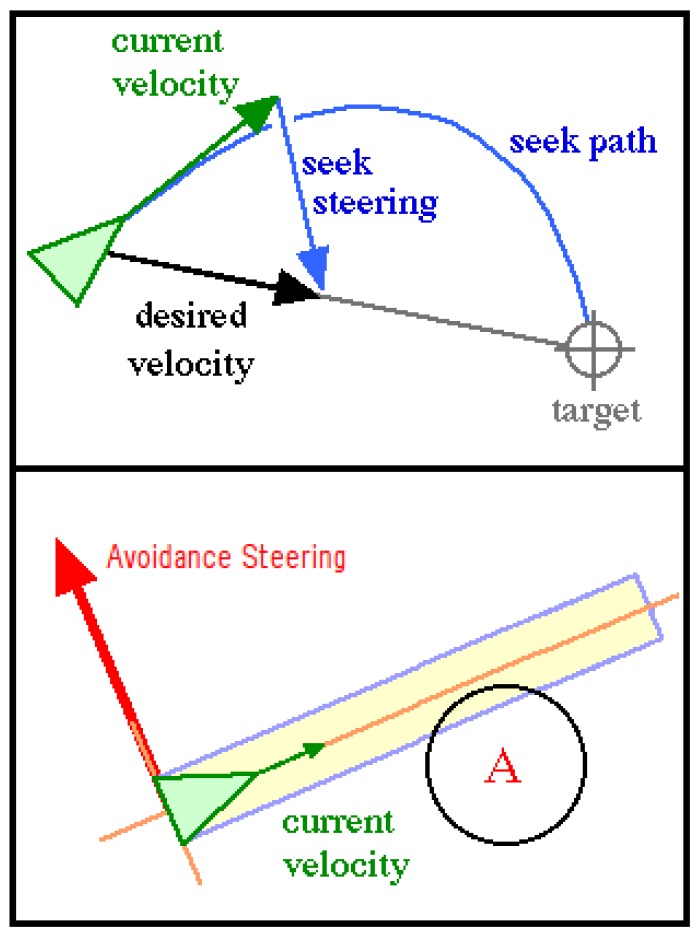
Steering Behaviors. Two Behaviors (Seek and Avoid) and their corresponding vectors are shown.

The seeking behavior tries to steer the vehicle towards a pre-defined position in the global coordinate system. The behavior adjusts the UGV velocity so it is aligned towards the target; the desired velocity is a vector defined from the vehicle position to the target at the maximum speed, and the steering vector is the difference between this desired velocity and the UGV current velocity. If only seeking behavior is used, the UGV will pass through the target and then turn back. The arriving behavior is similar to the seeking, but it implements a braking force when the vehicle is within a pre-defined distance to the target, thus making the UGV stop when it arrives at it. Figure 5 shows the Seek and Avoid behaviors, with their resulting steering vectors and the auxiliar items used to calculate them.

The avoidance behavior is intended to provide the vehicle with the ability to maneuver in an environment where obstacles may appear; it takes action only when an obstacle is detected in front to the UGV, meaning that if it is moving parallel to a wall, the avoidance will take no action. The basic algorithm assumes that both the vehicle and the obstacles are spheres, although variations can be made to take into account the shape, and then extends the bounding sphere of the UGV to create an imaginary cylinder lying along the forward axis of the UGV, the length of the cylinder depends on the speed of the vehicle and its ability to steer. The algorithm then calculates if any of the given obstacles intersect with the UGV's cylinder. If there is no collision, a zero vector is returned; if a collision is found, the center of the obstacle is projected on the side axis of the vehicle and a steer in the opposite direction is generated. If two or more obstacles are found, the one nearest to the vehicle prevails over the rest. The two behaviors (seek and avoid) can be combined by adding the steering forces resulting for each one. It is also possible to give more priority to one or another by performing a weighted addition.

## 6. Experiments

**Figure 6.**
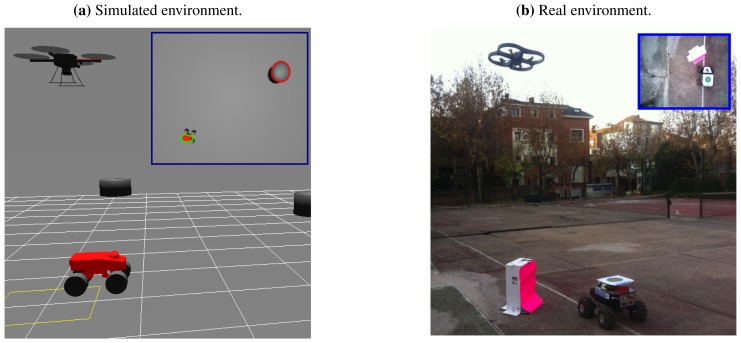
UAV and UGV in the proposed environments (**a**) Simulated environment. (**b**) Real environment. The blue squares in each figure represent the image acquired from the camera on board the aerial robot.

This section presents the results obtained with the proposed aerial–ground system. The approaches described in the previous sections have been implemented first on a simulation environment, and then with real robots. The software architecture used allows to use the same implementation in both environments, but it was necessary to use a realistic simulation of the entire system. First, a quad-rotor aerial robot model was used, as well as a skid-steering ground robot. Also, the sensors simulations that include typical error sources were added to the both robot models, as well as the camera model on board the UAV. By doing this, we ensure that the simulations and the real results are consistent, and that the algorithms can be translated to the real robots. The simulated and real environments are shown in Figure 6.

### 6.1. Simulations

Two main tests have been performed on the simulated environment. The first one consists of a single obstacle detection and avoidance maneuver, it is done to check each part of the solution proposed, and thus validate it.

**Figure 7.**
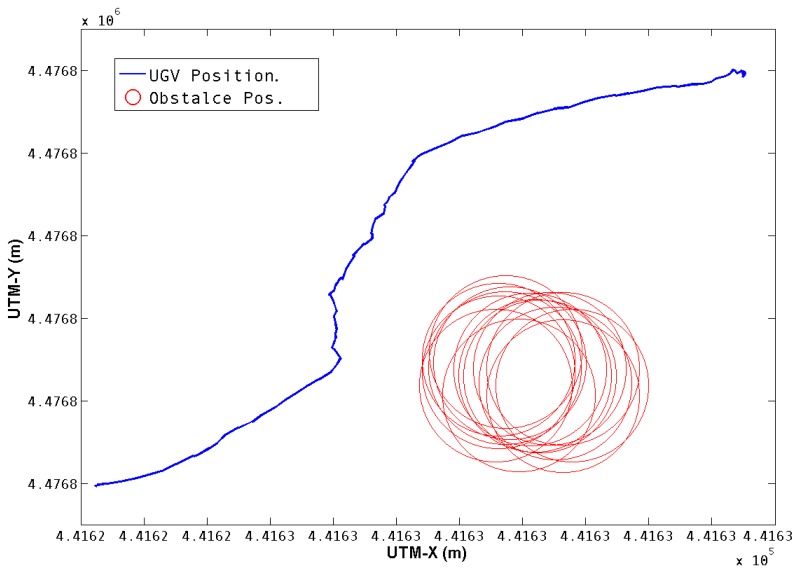
Single obstacle Avoidance. The trajectory of the UGV while performing the avoidance maneuver, and the detected obstacle positions are shown.

Figure 7 shows the trajectory performed for the first test. The position of the UGV in UTMs is obtained from the Extended Kalman Filter. The position of the obstacle is calculated from the aerial image and the corresponding transformations, as was described in Section 3. The total distance covered was 16.9 meters and was executed in 25 seconds.

**Figure 8.**
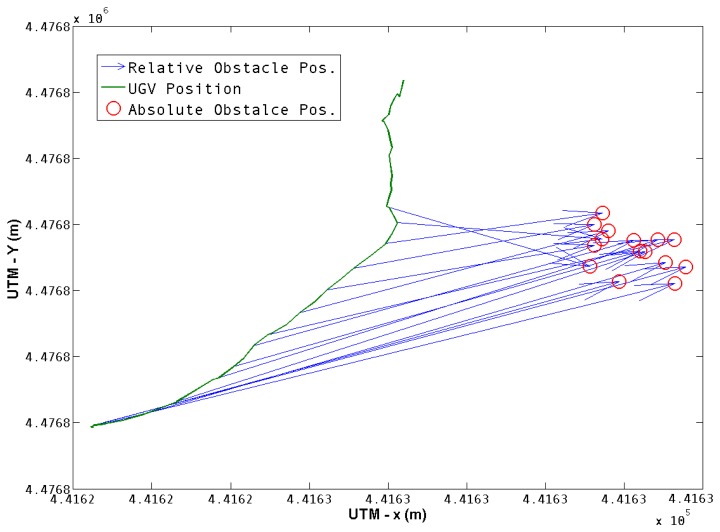
Obstacle Position Estimation. The green line denotes the UGV position. The blue arrows determine the transformation from each robot position to the center of the obstacle marked with red circles.

Figure 8 shows how each position was obtained, a transformation from the UGV position to the obstacle position is obtained according to Equation (4) and is represented by blue arrows. The resulting obstacle position in the world coordinate frame is represented by red circles. Once the obstacle is left behind, its position is no longer of interest, nor is it taken into account. It can be observed that there is a dispersal of the observations of the obstacle position, so it is of interest to have a characterization of the resulting positions of the obstacle.

**Figure 9.**
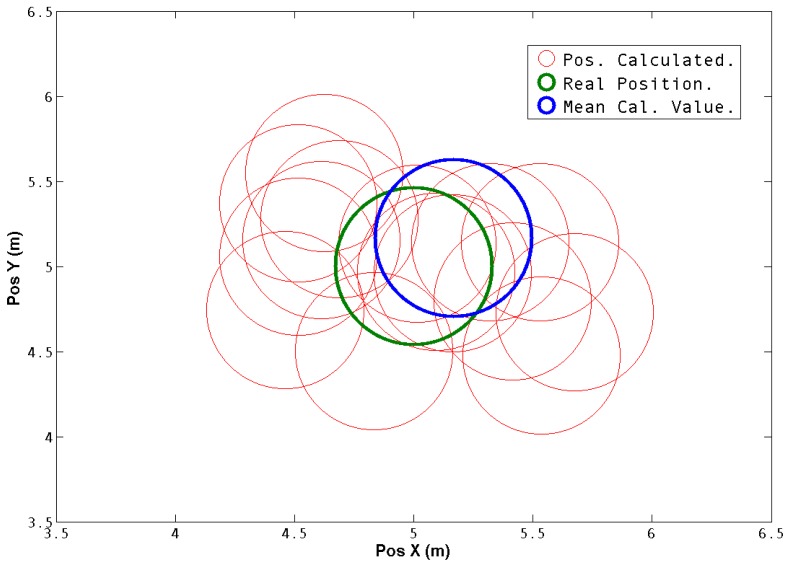
Obstacle Position (Real *vs*. Estimated). The red circles denote the estimated obstacle position. The green one shows the real position of the obstacle and the blue is the mean value of the calculated positions. All positions were translated to a common reference frame.

In Figure 9 the real obstacle position and the positions obtained with the system are translated to a common reference frame and compared. The mean square error and the standard deviation were calculated and are shown in Table 1. It is possible to see that both values are in good error ranges. The mean square error is less than 20% of the diameter of the obstacle, which is 1 meter, in the *X* axis and 30% on the *Y* axis.

**Figure 10.**
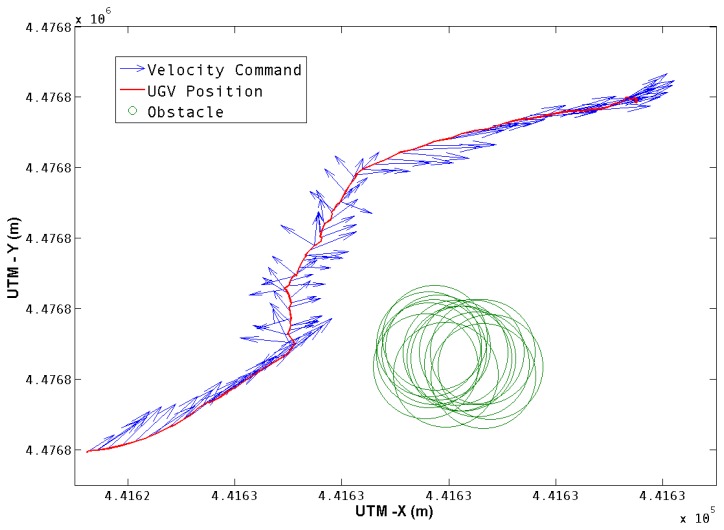
Velocity Commands. The red line describes the UGV trajectory, and the blue arrows represent the velocity commands at each position.

Once the obstacle position is obtained, the navigation algorithm uses this information to perform the avoidance maneuver according to the seek and avoid behaviors described in Section 5.2. The outcome of this navigation algorithm is the velocity command in each time step. The command is translated to the UGV reference frame, converted to linear and angular velocity, and sent to the robot's control system. Figure 10 shows the velocity commands generated for this test, together with the UGV's trajectory and the obstacle position.

**Figure 11.**
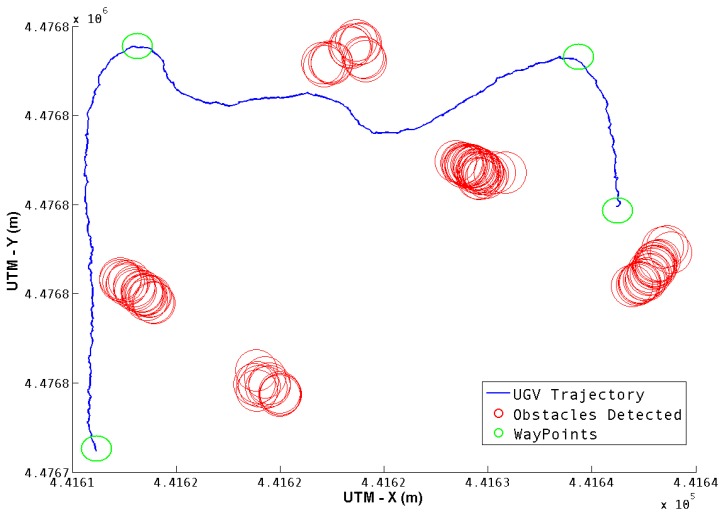
Second Test Trajectory. The blue line describes the UGV trajectory. The red circles represent the obstacles found in the pathway and the green circles are the way-points.

For the second test, three way-points were defined, in an environment with six cylindrical obstacles. While visiting the way-points, some obstacles were detected, and the corresponding avoidance was executed if necessary. Figure 11 shows the full trajectory, as well as the obstacles detected. Five of the six obstacles were detected, and all three way-points were reached successfully.

The position is given in UTM coordinates, as was explained in Section 4.2. Since the values obtained for the standard deviation are in all cases less than one meter, it can be concluded that this data gives a set of obstacle positions can be used for the navigation system. Table 2 shows the mean value and standard deviation of the five obstacles detected during the test.

### 6.2. Real Environment

A set of tests were designed and implemented in order to check the feasibility of the system outside of the simulated environment. The first test was oriented to check the inter-process communications. Therefore, a set of hovering flights were performed and data both from the UGV and the UAV was acquired in real-time using the middle-ware and software architecture developed for this system [21].

**Figure 12.**
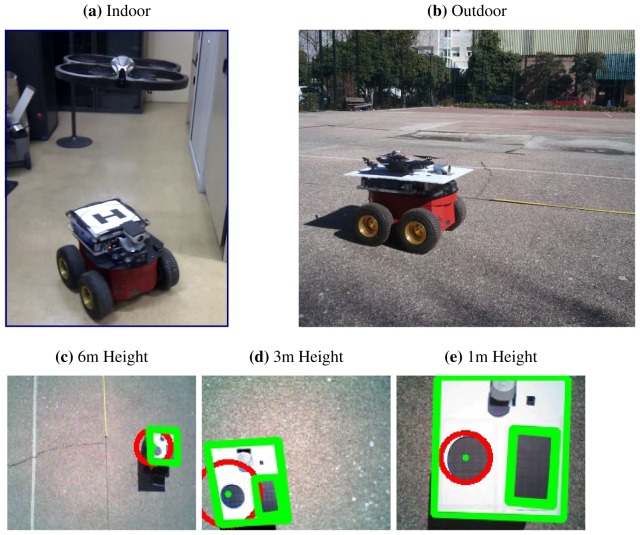
Initial Tests with real robots: (**a,b**) Communication and hovering. (**c**), (**d**) and (**e**) Aerial Images captured at different heights.

The second test was carried out in an outdoor environment. Its objectives was to obtain real images from the UAV and test the feature extraction algorithms. To facilitate the tests, a platform with a geometric shape pattern was shipped in the UGV. The platform designed have two main capabilities: it can be used for take-off and emergency landings, and it can also transport the UAV in case it ran out of battery. An additional advantage is that the platform design made it easier to track the UGV and distinguish it from other features on the ground. Finally, the navigation algorithm for the UGV was tested using a fixed target and a virtual obstacle position. A set of images from those previous tests are shown in Figure 12.

During the tests described previously, the image acquisition rate and processing times, likewise the telemetry data from both the UGV and the UAV, were tested and measured. At last, all the data processing and the commands sent to the navigation system were also measured in terms of its time period. The results of all those measurements are shown in Table 3.

It can be observed that the image streaming runs with an average frequency of 10Hz, and that all the other times or frequencies are faster than that value. In accordance with the software architecture (defined in [21]), the UGV's control and navigation systems run in the UGV on-board computer, and the image acquisition and processing is done on the base-station PC. Also, the inter-process communication core is handled by the ROS middle-ware framework, which enables the system to work at the same frequency as the image streaming. The results from those previous tests shown that the communications and image processing algorithms are feasible enough to perform obstacle-free navigation in real time.

In order to accomplish the last experiment with the full aerial–ground system. A set of obstacles were placed in different positions of the robot workspace. Then, a set of pre-defined and fixed targets were given to the UGV ensuring that to arrive there it must avoid the obstacles previously defined.

**Figure 13.**
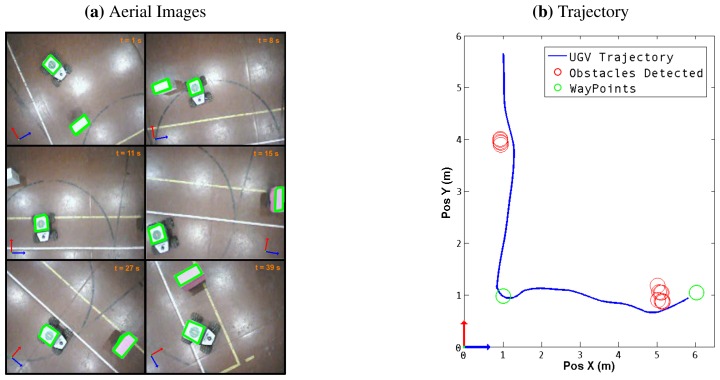
Field tests: (**a**) Sequence of images obtained from the UAV while performing an obstacle-avoidance maneuver. The orientation is represented with a blue and red arrow for the *X* and *Y* axes. (**b**) The UGV trajectory read from the EKF is shown in blue, additional marks for the target and the obstacle have been added.

The obstacle-avoidance maneuver was executed using the steering behaviors algorithm. The UAV was manually controlled in hovering mode over the UGV, and images from the aerial vehicle were acquired and processed using the feature extraction algorithm. The position of the UGV and the obstacle were extracted from the image and the information of the obstacles position sent to the UGV navigation system. Figure 13 shows a sequence of aerial images obtained during a trajectory, with the objects identified in each frame, it also shows the trajectory obtained from the odometry of the UGV The variables measured during the experiment are shown in Table 4.

The obstacles were successfully detected and avoided using the proposed system; the mean position and standard deviation of the observations are shown in Table 5. It should be emphasized that the error in the estimated position of the obstacles was less than 0.15*meters* in the last experiment. Moreover, the UGV maintained a mean safe distance from the obstacles of 0.3 meters. Therefore, despite the obstacles' position error, the UGV is unlikely to collide due to the safe distance between the obstacles.

## 7. Concluding Remarks

A hybrid robotic system has been designed and implemented in order to provide a safe navigation system for a UGV, using the aerial image from a camera on board the UAV as the only source of information about the environment. The system is able to perform local real-time navigation and exploration in large semi-structured environments; it can also build near-accurate maps with the absolute position of the obstacles found it its path. These maps can be used to generate local path planning or be fed back to other robots or mission planners. The geographic positions from the obstacles are obtained through an original fusion method employing both the real-time aerial image and the UGV absolute position provided by the GPS.

The robustness of the system was checked according to the mission requirements. Therefore, a set of experiments were done both in a simulation environment and an outdoor scenario with real robots. The ground robot moved around unknown and cluttered environments without colliding.

The results obtained are encouraging to continue researching and extend the potential of this collaborative robotic system.

## Figures and Tables

**Table 1.** *Mean Square Error* and *Standard Deviation* of the calculated obstacle position.

	*Mean Square Error (m)*	*Standard Deviation*
*X Axis*	0.1954	0.4236
*Y Axis*	0.3333	0.3053

**Table 2.** *Mean Value* and *Standard Deviation* for all the obstacles found in the Test Trajectory.

	*Mean Pos X (m)*	*Std. Dev. X*	*Mean Pos Y (m)*	*Std. Dev. Y*
*Obstacle 1*	441637.8	0.4743	4476756.3	0.6360
*Obstacle 2*	441623.3	0.7489	4476768.4	0.5152
*Obstacle 3*	441629.4	0.4939	4476761.9	0.2612
*Obstacle 4*	441613.1	0.5246	4476755.1	0.4076
*Obstacle 5*	441619.3	0.4924	4476749.7	0.3820

**Table 3.** Frequencies and Time Consumption for image and data streaming and processing.

	*Average Frequency (Hz)*	*Max. Period (s)*	*Min. Period (s)*
*Image Streaming*	10	0.446	0.003
*UAV Telemetry*	13	0.404	0.001
*UGV Telemetry*	20	0.051	0.049
*UGV Navigation System*	10	0.103	0.096
*Image and Data Processing*	37	0.102	0.010

**Table 4.** Trajectory Parameters and Additional Information.

*Trajectory Time:*	39 *s*
*UGV Max Speed*	0.3 *m*/*s*
*UGV Mean Speed*	0.2048 *m*/*s*
*UAV Max altitude*	4.812 *m*
*UAV Mean altitude*	4.51 *m*
*Total Trajectory Distance:*	10.1999 *m*

**Table 5.** *Mean Value* and *Standard Deviation* for the obstacles found in the trajectory.

	*Mean Pos X (m)*	*Std. Dev. X*	*Mean Pos Y (m)*	*Std. Dev. Y*
*Obstacle 1*	0.9896	0.0194	4.1373	0.0468
*Obstacle 2*	5.0930	0.1143	1.1158	0.0492
